# Susceptibility of periodontal pathogens to a novel target-specific drug delivery system containing self-nanoemulsifying curcumin: An in vitro study

**DOI:** 10.34172/japid.2023.024

**Published:** 2023-12-06

**Authors:** Veena HR, Riya Achamma Daniel, Ashwin Prabhu, Shilpa P, Suman Basavaraju

**Affiliations:** ^1^Department of Periodontology, KLE Society’s Institute of Dental Sciences, Bengaluru, Karnataka; ^2^Department of Dental Surgery, MIOT International, Chennai, India; ^3^KLE Society’s Institute of Dental Sciences, Bengaluru, Karnataka; ^4^Department of Periodontology, JSS Dental College & Hospital, Mysuru, India

**Keywords:** Antibacterial agents, Curcumin, Nanoparticles, Periodontitis

## Abstract

**Background.:**

Long-term use of many classic chemotherapeutic agents as adjuncts in the management of periodontitis has adverse complications, leading to seeking out naturopathic remedies. Although curcumin has been investigated in managing periodontitis, its therapeutic benefits have not been fully explored due to its limited solubility in an aqueous medium. This study aimed to develop a novel target-specific drug delivery system containing 1% self-nanoemulsifying curcumin (SNEC) in a hydroxypropylmethylcellulose (HPMC) matrix and evaluate the susceptibility of periodontal pathogens to this system in vitro.

**Methods.:**

Its antibacterial activity against Tannerella forsythia, Porphyromonas gingivalis, Prevotella intermedia, and Aggregatibacter actinomycetemcomitans was evaluated and compared to pure nano-curcumin and SNEC alone by estimating their minimum inhibitory concentrations (MIC).

**Results.:**

The antibacterial activity of pure nano-curcumin, SNEC, and SNEC in HPMC against the four periodontal pathogens evaluated in terms of MIC was recorded in the range of 0.2‒0.4, 0.4‒0.8, and 0.2‒0.8 µg/mL, respectively. However, the MIC of all three curcumin formulations against the periodontal pathogens tested was higher than that of the standard moxifloxacin. While both pure nano-curcumin and SNEC showed increasing values of inhibition zones with increasing concentrations on disk diffusion assay, lower concentrations of SNEC in HPMC did not show a zone of inhibition against the tested pathogens.

**Conclusion.:**

The novel delivery system containing SNEC in HPMC may be a potential adjunct in managing periodontitis due to its probable sustained antimicrobial activity against the tested periodontal pathogens.

## Introduction

 Periodontitis is an immuno-inflammatory disease of the periodontium, manifested as irreversible destruction of the tooth-supporting apparatus following an alteration in its integrity in response to bacterial biofilm. Periodontitis was managed as an infectious disease with the recognition of its microbial etiology in the 1960s and 1970s. The standard periodontal therapy, i.e., mechanical debridement, is a highly demanding procedure involving adjunctive antimicrobial agents.^1^ Subsequent identification of host immune response in the 1980s introduced the strategy of host modulation therapy (HMT).^2^ Effective administration of these perioceutics into the periodontal pocket requires an adequate drug concentration at the target site and a means to maintain this concentration for a prolonged duration.^3^ The development of novel carrier systems for targeted delivery of these pharmacological agents has shown promise by crucial improvements in the pharmacokinetics of the active therapeutic agent. Nano-drug delivery systems are a relatively new and rapidly developing science where materials in the nanoscale range (1‒100 nm) are employed to deliver therapeutic agents to specific targeted sites in a controlled manner.^4^

 Long-term use of many of these tested classic agents has adverse complications, leading to seeking out naturopathic remedies. In dentistry, 73% of the new antibacterials approved by the US Food and Drug Administration (US FDA) are phytotherapeutics or plant-derived medications. In systemically healthy patients with periodontitis, the local use of phytotherapeutics as adjuncts to scaling and root planing (SRP) has promoted additional improvements in clinical parameters.^5^

 Turmeric, or *Curcuma longa*, has been traditionally exploited for its medicinal properties since time immemorial. Its curcumin component possesses both antibacterial and anti-inflammatory properties, targeting both microbial plaque and host response, among a myriad of other medicinal properties.^6^ Although curcumin has been investigated in managing periodontitis, its clinical benefits have not been fully explored due to its limited solubility in an aqueous medium.^7^ Numerous strategies have been employed to improve curcumin’s cellular uptake and bioavailability. Self-nanoemulsifying drug delivery systems (SNEDDS) have been shown to possess superior emulsification ability, solubility, permeability, and entrapment efficiency for topical and site-specific delivery in managing skin and other local pathologies, respectively.^8,9^ Hydroxypropylmethylcellulose (HPMC), as a biodegradable matrix carrier, is considered desirable for sustainable delivery of the self-nanoemulsifying curcumin (SNEC) for up to 3 hours compared to the sustainability of the formulation devoid of the matrix.^10^

 Thus, this study is the first of its kind to develop a target-specific drug delivery system containing 1% SNEC in an HPMC matrix and evaluate its antibacterial activity in comparison to pure nano curcumin by estimating their minimum inhibitory concentration (MIC) in vitro against four periodontopathogens*, Tannerella forsythia* (*Tf*), *Porphyromonas gingivalis* (*Pg*), *Prevotella intermedia* (*Pi*) and *Aggregatibacter actinomycetemcomitans* (*Aa*).

## Methods

 Following approval from the institutional review board, pure nano-curcumin was procured from Beloor Bayir Biotech Ltd. (Batch no: BBB/NHC/B/17010). A commercially available SNEC system (SNEC 30 capsules; US Patent No: US8835509; Arbro Pharmaceuticals Pvt. Ltd.: ISO 9001:2008 Certification and ISO/IC 17025:2005 Accreditation) was also procured. The novel target-specific drug delivery system containing 1% SNEC in the HPMC matrix was developed in the Department of Pharmaceutics of the College of Pharmacy in Bangalore.

**Figure 1:**
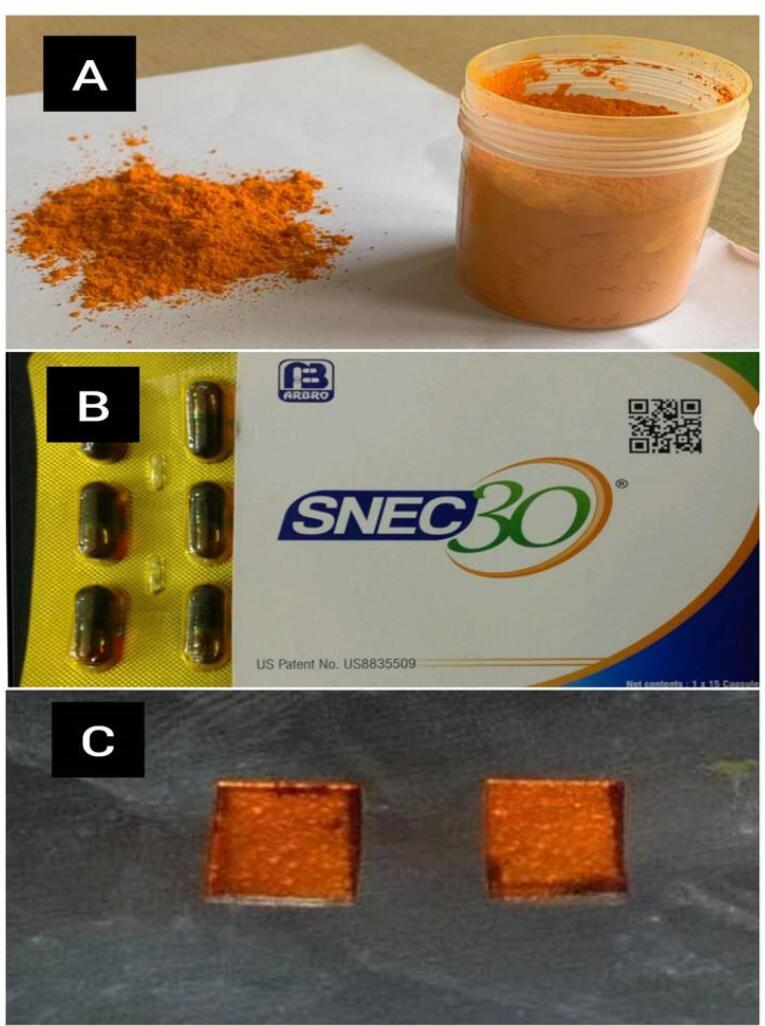
Test drug agents (a) pure nano-curcumin, (b) SNEC, and (c) 1% SNEC in HPMC

 The in vitro antibacterial activity of the following drug solutions (Figure 1) was determined by estimating each of their MIC against *Tf* (ATCC 43037), *Pg*(ATCC 33277), *Pi* (ATCC 25611), and *Aa* (ATCC 43718).

- Pure nano-curcumin
- SNEC
- 1% SNEC in HPMC

###  MIC 

**Figure 2:**
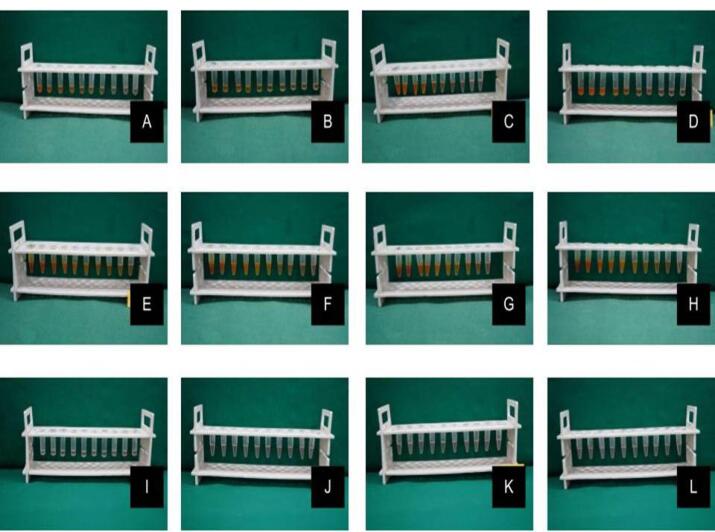
MIC estimation for (A) pure nano-curcumin against *Tf*, (B) pure nano-curcumin against *Pg*, (C) pure nano-curcumin against *Pi*, (D) pure nano-curcumin against *Aa*, (E) SNEC against *Tf*, (F) SNEC against *Pg*, (G) SNEC against *Pi*, (H) SNEC against *Aa*, (I) SNEC + HPMC against *Tf*, (J) SNEC + HPMC against *Pg*, (K) SNEC + HPMC against *Pi* and (L) SNEC + HPMC against *Aa*

 The stock solution of the test drugs was prepared by dissolving it in dimethylsulfoxide to ensure good solubilization. For the MIC assay, nine serial dilutions of each drug solution were formulated in thioglycollate broth to obtain the following concentrations: 100, 50, 25, 12.5, 6.25, 3.12, 1.6, 0.8, 0.4, and 0.2 µg/mL. From the maintained stock cultures of required organisms, 5 µL was taken and added to 2 mL of thioglycollate broth. In each of the 10 tubes of each drug solution, 200 µL of the above culture suspension of a particular pathogen was added. The tubes were incubated for 48‒72 hours in an anaerobic jar at 37 °C and observed for turbidity. This procedure was repeated for each of the tested organisms (Figure 2).^11^

###  Disk Diffusion Test

**Figure 3:**
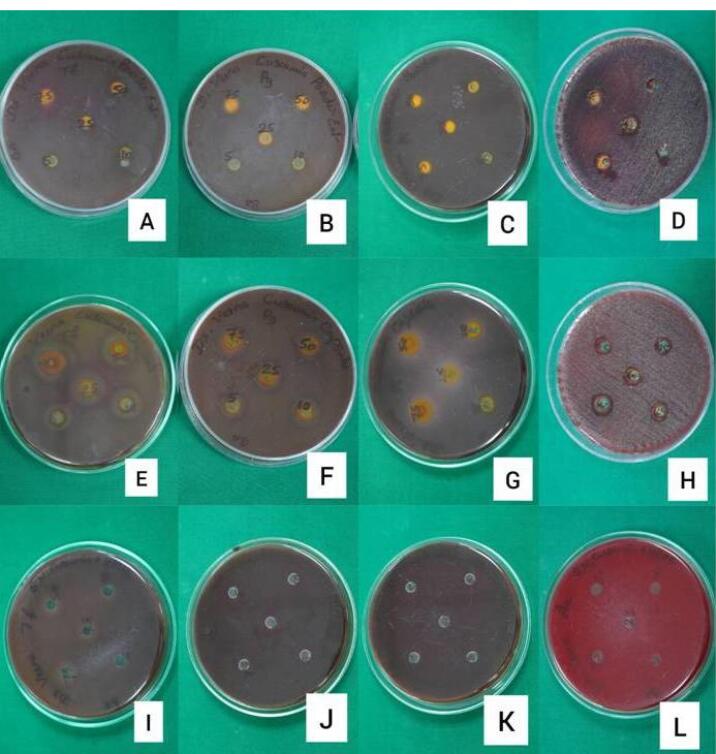
Disk diffusion assay of (A) pure nano-curcumin against *Tf*, (B) pure nano-curcumin against *Pg*, (C) pure nano-curcumin against *Pi*, (D) pure nano-curcumin against *Aa*, (E) SNEC against *Tf*, (F) SNEC against *Pg*, (G) SNEC against *Pi*, (H) SNEC against *Aa*, (I) SNEC + HPMC against *Tf*, (J) SNEC + HPMC against *Pg*, (K) SNEC + HPMC against *Pi* and (L) SNEC + HPMC against *Aa*

 The suspensions of the tested organisms’ suspensions were visually adjusted with broth to equal that of an 0.5 McFarland turbidity standard that had been vortexed. The suspensions were standardized with a photometric device. Within 15 minutes of adjusting the inoculum to an 0.5 McFarland turbidity standard, a sterile cotton swab was dipped into the inoculum and rotated against the tube wall above the liquid to remove excess inoculum. The entire surface of the agar plate was swabbed three times, rotating the plates approximately 60º between streaking to ensure even distribution. Care was taken to avoid contacting the sides of the plates and creating aerosols. The inoculated plate was allowed to stand for at least 3 minutes but not longer than 15 minutes before creating the wells. A hollow tube of 5 mm diameter was heated, pressed against the inoculated agar plate, and removed immediately by making a well in the plate. Likewise, five wells were made on each plate. With the help of a micropipette, concentrations of 75, 50, 25, 10, and 5 µL/mL of each drug solution were added to individual wells. The plates were incubated within 15 minutes of drug application. The plates were inverted, stacked no more than five plates high, and incubated for 18‒24 hours at 37 ºC in an incubator. The plates were read-only if the microorganisms’ growth lawn was confluent or nearly confluent, and the diameter of the inhibition zone was measured to the nearest whole mm by holding the measuring device (Figure 3).^11^ The mean values of assays performed in triplicates were recorded.

## Results

###  MIC 

 The results of MIC were tabulated for each tested organism against each of the serially diluted concentrations of each of the tested formulations of curcumin (Table 1). Against*Tf*, SNEC in HPMC showed an MIC higher than the comparable values of pure nano-curcumin and SNEC. Against *Pg*, pure nano-curcumin showed a lower MIC compared to both SNEC and SNEC in HPMC, the latter two having shown similar values. Against *Pi* and *Aa*, SNEC showed a higher MIC compared to the similar values observed with both pure nano-curcumin and SNEC in HPMC. However, the MIC of all tested formulations against the tested periodontal pathogens was higher than that of the standard moxifloxacin.

**Table 1:** Minimum inhibitory concentrations

**Organism**	**Sl. No.**	**Samples**	**100 µg/mL**	**50 µg/mL**	**25 µg/mL**	**12.5 µg/mL**	**6.25 µg/mL**	**3.12 µg/mL**	**1.6 µg/mL**	**0.8 µg/mL**	**0.4 µg/mL**	**0.2 µg/mL**
*Tf*	1	Pure nano curcumin	S	S	S	S	S	S	S	S	S	R
	2	SNEC	S	S	S	S	S	S	S	S	S	R
	3	SNEC + HPMC	S	S	S	S	S	S	S	S	R	R
*Pg*	1	Pure nano curcumin	S	S	S	S	S	S	S	S	S	S
	2	SNEC	S	S	S	S	S	S	S	S	R	R
	3	SNEC + HPMC	S	S	S	S	S	S	S	S	R	R
*Pi*	1	Pure nano curcumin	S	S	S	S	S	S	S	S	S	R
	2	SNEC	S	S	S	S	S	S	S	S	R	R
	3	SNEC + HPMC	S	S	S	S	S	S	S	S	S	R
*Aa*	1	Pure nano curcumin	S	S	S	S	S	S	S	S	S	S
	2	SNEC	S	S	S	S	S	S	S	S	S	R
	3	SNEC + HPMC	S	S	S	S	S	S	S	S	S	S

*Note*: S: Sensitive, R: Resistant. Standard values for MIC *Tf*: Moxifloxacin < 0.125 µg/mL *Pg*: Moxifloxacin < 0.125 µg/mL *Pi*: Moxifloxacin < 0.125 µg/mL *Aa*: Moxifloxacin < 0.125 µg/mL.

###  Disk diffusion

 The zones of inhibition observed for each tested organism against the different concentrations of each tested curcumin formulation were tabulated (Table 2). The inhibition zones for all pathogens around pure nano-curcumin showed a decreasing trend with lowered concentrations. In the case of SNEC, a similar pattern was observed for all organisms except *Aa.* The zone of inhibition was lesser for both pure nano-curcumin and SNEC compared to the standard moxifloxacin. All the tested pathogens, however, seemed to be resistant to the lower concentrations of the system containing SNEC in HPMC with no signs of a zone of inhibition.

**Table 2:** Disk diffusion assay

**Organism**	**Sl. No**.	**Samples**	**75 µL/mL**	**50 µL/mL**	**25 µL/mL**	**10 µL/mL**	**5 µL/mL**
*Tf*	1	Pure nano-curcumin	28 mm	25 mm	23 mm	20 mm	18 mm
	2	SNEC	25 mm	20 mm	20 mm	18 mm	15 mm
	3	SNEC + HPMC	10 mm	R	R	R	R
*Pg*	1	Pure nano-curcumin	25 mm	20 mm	18 mm	13 mm	12 mm
	2	SNEC	20 mm	18 mm	15 mm	15 mm	13 mm
	3	SNEC + HPMC	10 mm	R	R	R	R
*Pi*	1	Pure nano-curcumin	18 mm	15 mm	13 mm	10 mm	8 mm
	2	SNEC	15 mm	13 mm	12 mm	10 mm	8 mm
	3	SNEC + HPMC	12 mm	R	R	R	R
*Aa*	1	Pure nano-curcumin	25 mm	18 mm	15 mm	R	R
	2	SNEC	30 mm	28 mm	23 mm	20 mm	15 mm
	3	SNEC + HPMC	12 mm	10 mm	R	R	R

Standard values for disk diffusion *Tf:* Moxifloxacin- 35 mm *Pg:* Moxifloxacin-35 mm *Pi:* Moxifloxacin- 35 mm *Aa:* Moxifloxacin-35 mm.

## Discussion

 With the primary etiology of periodontal disease being plaque bacteria and their byproducts, mechanical and chemical approaches targeting these periodontopathogens have largely been employed in the management.^1^

 Phytotherapy is potentially effective in comparison to modern medications as an adjunct to SRP procedures in the management of periodontitis.^12^ Curcumin is one such herbal derivative with proven antibacterial, antioxidant, anti-inflammatory, antiproliferative, pro-apoptotic, and anticancer properties.^6^ The safety of curcumin has been well established by human studies, which revealed that oral doses of up to 12 gm/day are well tolerated.^12^ While it is categorized as ‘Generally Recognized As Safe’ by the US FDA, there is substantial evidence about the safety of life-long ingestion of about 100 mg/day in the Indian population.^6^ Nanoparticles of curcumin administered orally in rats for 14 days were safe at a single dose of 2 mg/kg.^13^ Curcumin, as recorded by a systematic review by Fakheran et al^14^ could promote periodontal health not only by inhibiting the periodontopathogens but also by modulating the host immune response. The systematic review also stated that the antibiofilm property of curcumin is responsible for its antibacterial activity.^14^ Clinical studies by Behal et al,^15^ Mali et al,^16^ Gottumukkala et al,^17^ Bhatia et al,^18^ and Nagasri et al^19^ found that adjuvant topical curcumin administration helps improve the microbiological parameters in the management of periodontitis.However, clinical trials that compared curcumin with chlorhexidine showed contradictory results, with few studies showing comparable microbiological outcomes^16,17^ and some showing chlorhexidine to be superior^20^ and other curcumin to be superior.^21^ Another systematic review and meta-analysis by Zhang et al,^22^ however, concluded that while curcumin results in comparable clinical outcomes similar to chlorhexidine, further research was mandated to firmly establish the clinical efficacy of curcumin. Thus, while curcumin produced mild to moderate beneficial effects as an adjunct to SRP, further research was to be aimed at improving the substantivity of the drug and also to prevent early recolonization of periodontal pathogens.^17^

 de Oliveira et al^23^ carried out a systematic review, reporting that the route of administration of a phytochemical is an important parameter that influences its efficacy by interfering with the bioavailability required to promote particular biological effects. Local drug delivery allows targeted treatment for periodontitis, helps achieve the required drug concentration significantly above the MIC of periodontopathogens, maintains it over an adequate time, and reduces the overall dosage administered.^24^ Several herbal extracts and synthetic drugs have been encapsulated into nano-delivery systems for targeting the periodontal pocket.^4^

 Like any other phytopolyphenol, curcumin is poorly soluble in water and must be solubilized in solvents like ethanol.^25^ Promising approaches to increase curcumin’s therapeutic efficiency include using structural analogs, nanoparticles, liposomes, micelles, phospholipid complexes, and combinations with adjuvants like piperine for delivery.^25,26^ In vivo studies conducted in rats have revealed that SNEDDS containing curcumin have significantly elevated C max and bioavailability compared to an aqueous suspension of curcumin.^27^ The system is also safe and non-toxic, apart from the comparatively superior wound healing and anti-inflammatory properties.^8^ Previous research has resulted in developing SNEDDS of curcumin to enhance the solubility and skin permeation with elevated entrapment efficiency and good transdermal penetration ability.^8,9^

 The MICs of pure nano-curcumin, SNEC, and SNEC in HPMC tested against the four periodontal pathogens in this study were in the range of 0.2‒0.8 µg/mL. This range of MIC was found to be much lower than that of pure curcumin, as observed in a previous study where curcumin inhibited the formation and maturation of biofilm and reduced its metabolic activity.^28-31^ This may be attributed to the superior antibacterial properties of nanoparticle biomaterials apart from their ultra-small sizes, large surface-area-to-mass ratio, and unique physical and chemical properties.^4^ Results from an in vitro study by Rachmawati et al^32^ have suggested that the improved outcome of curcumin-loaded SNEDDS can be attributed to its enhanced solubility in the nanoemulsion system.

 Furthermore, in this study, the inhibition zones for all tested pathogens increased with increasing concentrations of curcumin formulations. This finding was in accordance with a previous work that tested the antimicrobial efficacy of pure curcumin formulation against *Pg*.^33^ It has also been previously recorded that curcumin, in a dose-dependent manner, prevented bacterial adhesion and biofilm formation.^31^

 However, a larger zone of inhibition in disk diffusion assay could also mean the leaching of antimicrobial agents into the surrounding environment, which could be partly attributed to a gross incompatibility between the test agent and its vehicle, decreasing its antimicrobial activity. Furthermore, incompatibility of the medium with the antimicrobial agent could prevent any visually appreciable zone of inhibition while protecting the drug itself.^34^ Despite a low MIC of SNEC in HPMC, the disk diffusion assay for SNEC in HPMC showed a narrow zone of inhibition for tested pathogens, which can be attributed to the properties of the novel targeted drug delivery system. The HPMC employed as a vehicle in our test drug system could act as a precipitation inhibitor to SNEDDS and control its drug release by making it supersaturated and enhancing its aqueous solubility and absorption to many folds for drugs.^8,9,10,35,36^ Another independent study that characterized in vitro, a formulation containing SNEC in HPMC concluded that HPMC maintained the supersaturation state of curcumin by developing hydrogen bonds with the latter and thus improving the drug solubilization and viscosity of the system, thereby enhancing the absorption and bioavailability of curcumin.^37^ It was observed in an in vitro study that the presence of 10% w/w of HPMC in the tested formulations resulted in almost 100-fold greater concentrations of curcumin compared to the formulation without the polymer. This high drug concentration was further found to be sustained for 3 hours in comparison to the formulation without the polymer.^10^

 While previous works of other researchers have shown pure nano-curcumin to be superior to pure curcumin, in this study, all formulations tested presented with antibacterial activity in the range of 0.2‒0.8 µg/mL. Although this study employed standard cultures and controlled lab environments to obtain standardized results, the statistical and clinical significance of the observations could not be evaluated. Furthermore, the drug release mechanisms and the substantivity and sustainability of the developed formulation are yet to be evaluated. Thus, future large-scale studies employing cultures from clinical samples of individuals in different periodontal health statuses need to be conducted in both in vitro and clinical setups with long-term and short-term follow-up periods along with the assessment of the pharmacokinetics for validating these observations before clinical implementation.

## Conclusion

 It can be concluded that the novel phytopharmaceutical targeted delivery system containing SNEC in HPMC, tested in this study, may serve as a potential adjunct in the management of periodontitis, owing to its probable sustained antimicrobial activity against the tested periodontal pathogens. However, future studies are mandated for the validation of the results of this study before its introduction into clinical practice.

## Acknowledgments

 The authors thank the Rajiv Gandhi University of Health Sciences, Bangalore, for funding this research project. The authors would also want to acknowledge the Department of Pharmaceutics, KLE Society’s Pharmacy College, Bengaluru, Karnataka, and Maratha Mandal’s Central Research Laboratory, Maratha Mandal’s NGH Institute of Dental Sciences & Research Centre for rendering their support for this study.

## Competing Interests

 The authors declare that they have no competing interests.

## Consent for Publication

 Not applicable.

## Data Availability Statement

 The datasets used and/or analyzed during the current study are available from the corresponding author upon reasonable request.

## Ethical Approval

 Not applicable.

## Funding

 The study was supported by a grant from the Rajiv Gandhi University of Health Sciences, Bangalore.
